# Dissertation on the Elevation of the Dental Profession

**Published:** 1845-09

**Authors:** E. Townsand


### ARTICLE III.

*Dissertation on the Elevation of the Dental Profession.* Read before the American Society of Dental Surgeons, at its Sixth Annual Meeting, held in the city of New York, August, 1845. By E. Townsand, D. D. S.

The question has often been asked, how shall we elevate the character of the profession, of which we are individually members; and what means shall we use, to raise its reputation to a height commensurate with its just claims, as a science of so much importance to the comfort of the human family?

Let me mention *one* of the means by which we can, each of us, do our part to attain this much desired end. It is by being, in the strictest sense of the word, honest. Now it is not necessary, perhaps, that an American dentist should write on his door, "No cheating here," as do Chinese shopkeepers; but it is of the *last* importance that our character, as a professional body, should be as far above all fear or suspicion of dishonesty, as its skill and success are beyond all doubt or dispute. If we had all, at all times, been strictly upright—avoiding deception and quackery in all its forms and branches—doing always the best our judgment suggested—without being swayed in one case by deficiency of compensation—in another, dismayed by difficulties not easily surmounted—or even yielding to the more amiable weakness, reluctance to inflict pain—it is certain the dental surgeon would at this time stand in a more enviable position, and the art he professes to practise and understand, be very differently estimated.

It is to be deplored, that so many leave other occupations, which they imagine less lucrative, and commence the practice of what *they* call dentistry, with little or no previous preparation or training; "taking it up," as they say, just as some persons speak of "getting religion," as though it were a thing to hold in the hand or put in the pocket; while others contrive to continue their original calling, and yet, as one told me not long since, "do a little dentisting," to eke out the less profitable occupation. Now such men as these are often well meaning, generally have some ingenuity, often just knowledge enough to do mischief; but they have not, and, in their narrow sphere of observation, cannot have, the slightest conception of the blunders they commit. They are really *not* aware of the extent of their dishonesty; but in dentistry, the saying of Napoleon, that "a blunder is *worse* than a crime," becomes a serious truth.

We will suppose another case. A young man applies to a respectable private practitioner, and wishes to be received into his office as a student. The practitioner is an honest man, and therefore the request is met, not by prompt admission, but by inquiries as to his previous preparation; and the answers to these inquiries show that he is a graduate of one of our universities, has been born and bred a gentleman, his head and heart well educated, and so far calculated to do honor to *any* calling; but his hands, they are totally ignorant; his fingers can wear rings and gloves, can even use a pen, but for tools he has a contempt which he hardly attempts to disguise. Yet he expects to be received as a student, and prepared in a few months to enter an office of his own. He is not received, however; the conscientious man to whom he has applied, recommends an apprenticeship to some mechanical trade, gunsmith, jeweller, watchmaker, any thing that will educate his hands—and so dismisses him. Alas! he has not far to go, before he finds one less scrupulous; and in six months, or perhaps less time, we may find him in a fashionable street, with a door plate as large as a newspaper, ready to fill, file and extract, with unequalled facility and despatch. If, by any chance, he succeeds in an operation, he is not half so much astonished as one would suppose—to succeed is to him a matter of course; if he fails, and mischief results, he says, as the boy did of his whistling, "it did itself."

Need I remark on the great want of uprightness and integrity which this case exemplifies; on the total absence of right feeling for the credit of the profession, in both—of enlightened policy for his own reputation, on the part of the teacher? for, if he was even disposed to forget that the public would hold *him*, in a measure, responsible for the ignorance and mismanagement of one he had professed to instruct, his experience must have made him fully aware of the countless mistakes and irreparable mischief, which his half taught scholar might perpetrate, with the best intentions in the world. But the almighty dollar was, to him, all in all, and never will our profession rise to its proper elevation, until the dollar is estimated at the dollar's worth—until the true dignity of labor is recognized, and we toil for higher ends than the "penny fee" of the old song. Labor is a law of our being; from it no one is, or can be, exempt; and since it is the thought, the feeling, with which we toil, which makes our labor degrading drudgery or ennobling occupation, every man should do whatever his hands find to do, not only with all his might, but with elevation of purpose, looking always to spiritual growth,

"Taking an impulse and vibration to an end beyond its own."

For the professional man, be he lawyer, doctor or dentist, is often so happy as to find himself toiling assiduously, in utter disregard and often entire forgetfulness of the mere money compensation. Which of you has not felt this? and let me tell you *no* one, however skilful and accomplished, who does not and cannot feel this unselfish delight in usefulness for its own excellent sake, ever will, even in the most worldly view of the case, rise to eminence in his profession. There is a chord in the public mind, ever responsive to true nobleness; even the low and vulgar *perceive* the superiority with which they do not sympathise. To the really good, we accord involuntary homage. The world has clearer eyes than we give it credit for, and soon sees whether a man is impelled by high or low motives of action. The false coin is rejected, the ring of the true metal recognized, and the great truth established, that "the minds of other men mirror back ourselves;" for, even as it is easy to judge of the character of a lawyer, by the class of clients who frequent his office—of the physician, by the class of patients who require his services—so will an enlightened, cultivated and *honest* dentist attract to his chair, those on whom it will be both an honor and pleasure to attend.

One bright example of the elevation of character which has been attained in our profession, I may be permitted to mention, if it were only to express reverential regret that its living worth no longer illuminates our ranks. I allude, as you will easily conceive, to Edward Hudson, equally great and good, and most emphatically honest.

> "None knew him but to love him,
> Or named him but to praise!"

If I dared to lift the veil from his private life, I could tell you with what patient industry he toiled, year after year, to accumulate the means to liquidate large claims, which had no legal, but a clear moral hold on his justice and sense of right. Or if this were a time to intrude *myself* upon your notice, I could tell you with what almost fatherly kindness he extended the hand of encouragement to me, when I entered the profession he adorned; enlightening my inexperience, fortifying me against self-distrust, and cheering me under depression, although I had not the slightest claim upon his valuable time and experience. When I forget him, "may my right hand forget its cunning."

Even as a straw will indicate the direction of the wind, the circumstance I am about to relate will show you the bias of his character. Some time ago, a woman whom I knew to be a servant, came to me to have some trifling operation on her teeth; on examining her mouth, I found a number of teeth so well filled, that I asked who had had the care of it. She told me Dr. Hudson had filled her teeth for her. He visited the family in which she served, saw her front teeth needed care, and told her to come to him and he would put them in order; and in what order I found them! perfect as the day the work was done, each bright little disk of gold glittered in the clean and healthy bone; and this he did, when his fame was at its height, when his minutes were worth dollars, and evidently he had bestowed as much care and attention on the mouth of that poor Irish girl, as if she could have remunerated him for his trouble, and appreciated the beauty of his work.

Another circumstance, as slight in itself, forcibly impressed me with his conscientiousness. I entered his office one day, and found him sitting with a book in his hand, as though he had no connection with this work-a-day work—and to find Dr. Hudson unemployed during business hours, was indeed a rarity. When I expressed my surprise, he said, "My young friend, I feel to-day, as I suppese all men feel at times, as if I could do nothing well, nothing as it should be done, and I have sent away four patients this morning, and six yesterday, because I was afraid I should not do them justice; and I prefer crowding *next* week, and additional fatigue, to ill-executed work this." This needs no comment.

The influence of such a man does not end with his life. His fame and worth are the heritage of all time. If it is not in the power of every man to become as distinguished for grace of manner and skilful execution as Edward Hudson, it is possible for all to be equally honest, and exercise, in a measure, and according to their worth, as beneficial an influence on all connected with them. Every thing is possible to a determined will; and a steady purpose, combined with good principles and integrity, will as certainly lead a man to eminence in our profession, as water finds its level. No man, however humble, is willing to feel or admit himself utterly insignificant. We all trust, that in some circle, however small, we have bearing, force and weight. Neither let any one imagine his merit underrated by the public; if there is an error in the estimation in which a man is held, nine times out of ten it is the other way. If we are really great, our greatness will be sufficiently manifest; the false often passes for the true—rarely, indeed, is the true mistaken for the false.

"Oh, Iole! how did you know that Hercules was a god?" "Because," answered Iole, "I was content when my eye fell on him. When I beheld Theseus, I desired that I might see him offer battle, or, at least, guide his horses in the chariot-race; but Hercules did not wait for a contest; he conquered whether he stood, or walked, or sat, or *whatever thing he did.*"

Let no one suppose, because his position is humble, his example is of little moment; the silent influence of character is immense. George Fox, the apostle of the Quakers, used to say, that "the example of an honest man would shake the country for ten miles round;" and one of the wisest men of the present day has said, "nothing so much astonishes men, as plain dealing and common sense."

My friends! I will intrude no longer on your time. I am aware I have told you nothing new, nothing but what any one of you could have said better than I have said it; it only remains for me to thank you for the patience with which you have listened to my suggestions, which I do, most sincerely.

